# The Impact of the Older Person’s Grant Expansion on Hypertension Among Older Men in Rural South Africa: Findings From the HAALSI Cohort

**DOI:** 10.1093/geroni/igae010

**Published:** 2024-02-08

**Authors:** Haeyoon Chang, Janet Jock, Molly S Rosenberg, Chihua Li, Tsai-Chin Cho, Thomas A Gaziano, Lynda Lisabeth, Lindsay C Kobayashi

**Affiliations:** Department of Epidemiology, School of Public Health, The University of Michigan, Ann Arbor, Michigan, USA; O’Neill School of Public and Environmental Affairs, Indiana University-Bloomington, Bloomington, Indiana, USA; Department of Epidemiology, School of Public Health, Indiana University, Bloomington, Indiana, USA; Institute of Social Research, University of Michigan, Ann Arbor, Michigan, USA; Department of Epidemiology, School of Public Health, The University of Michigan, Ann Arbor, Michigan, USA; Division of Cardiovascular Medicine, Brigham and Women’s Hospital, Boston, Massachusetts, USA; Department of Epidemiology, School of Public Health, The University of Michigan, Ann Arbor, Michigan, USA; Department of Epidemiology, School of Public Health, The University of Michigan, Ann Arbor, Michigan, USA

**Keywords:** Aging health, Pension, Rural health, Socioeconomic condition, South African Older Person’s Grant Policy

## Abstract

**Background and Objectives:**

Hypertension is a major modifiable contributor to disease burden in sub-Saharan Africa. We exploited an expansion to age eligibility for men in South Africa’s noncontributory public pension to assess the impact of pension eligibility on hypertension in a rural, low-income South African setting.

**Research Design and Methods:**

Data were from 1 247 men aged ≥60 in the population-representative Health and Aging in Africa: A Longitudinal Study of an INDEPTH Community in South Africa in 2014/2015. We identified cohorts of men from 0 (controls, aged ≥65 at pension expansion) through 5 years of additional pension eligibility based on their birth year. Using the modified Framingham Heart Study hypertension risk prediction model, and the Wand et al. model modified for the South African population, we estimated the difference in the probabilities of hypertension for men who benefitted from the pension expansion relative to the control. We conducted a negative control analysis among older women, who were not eligible for pension expansion, to assess the robustness of our findings.

**Results:**

Older men with 5 additional years of pension eligibility had a 6.9–8.1 percentage point greater probability of hypertension than expected without the pension expansion eligibility. After accounting for birth cohort effects through a negative control analysis involving older women reduced estimates to a 3.0–5.2 percentage point greater probability of hypertension than expected. We observed a mean 0.2 percentage point increase in the probability of hypertension per additional year of pension eligibility, but this trend was not statistically significant.

**Discussion and Implications:**

Although the Older Person’s Grant is important for improving the financial circumstances of older adults and their families in South Africa, expanded pension eligibility may have a small, negative short-term effect on hypertension among older men in this rural, South African setting.

**Translational Significance:** Noncontributory pensions alone may not be sufficient to improve cardiovascular health among rural South African men. Further long-term evaluation data are needed, including in other population groups and geographic settings. Results may inform policy decisions related to public pensions by defining the social contours of the association between improved financial conditions in later-life and cardiovascular health outcomes in sub-Saharan Africa.

Due to its rapidly aging population, cardiovascular disease (CVD) is becoming an increasingly urgent public health concern in South Africa (1–3). The 2017 Global Burden of Disease report cited hypertensive heart disease, stroke, and ischemic heart disease as the leading causes of CVD burden in South Africa: from 1990 to 2017, the all-age disability-adjusted life years for these diseases increased by 71.4%, 37.7%, and 51.1%, respectively (3). Hypertension is a key contributor to the high prevalence of these 3 diseases (3,4). Approximately 71% of adults over 60 years of age in South Africa are estimated to have hypertension, with women exhibiting a greater impact of the effect of age on hypertension risk compared to men (5,6). At least one third of older men and women have not received a diagnosis, and, at most, 25% are receiving medication (5). The prevalence of undiagnosed and uncontrolled high hypertension in South Africa is a challenge that many health systems cannot currently address, especially in resource-constrained regions such as rural South Africa (5,7,8).

The Older Person’s Grant (OPG), formerly known as the Old Age Pension, is a vital social protection policy for older adults in South Africa, which may serve to improve population health in addition to its demonstrated effects on the alleviation of poverty (9,10). During Apartheid from 1948 to the early 1990s, the government designated many rural regions of South Africa as “homelands,” which were regions of forced racial and ethnic segregation (11). This policy left a majority share of the country’s population without basic health care, access to education, poor living standards, and without any public pension distribution (11,12). In 1993, the OPG became universal and noncontributory, with women eligible at age 60 and men at age 65 (9). As one of the most substantial public social assistance programs in South Africa, the OPG plays a crucial role in the redistribution of resources to poorer households and communities in an unequal and aging society (9). Through its impact on the social determinants of health, the OPG thus has the potential to directly address health disparities among older adults in rural South Africa.

In 2008, the OPG underwent a significant expansion to promote gender equality in the retirement age. Men’s retirement age was lowered incrementally from 65 to 60 years from April 2008 to April 2010 to match the women’s retirement age (13,14). Specifically, the men’s pension eligibility age was lowered from 65 to 64 in 2008, from 64 to 61 in 2009, and from 61 to 60 in 2010 (13,14). This pension expansion policy created exogenous variation in pension income among older men but not older women, providing an opportunity to evaluate the impact of pension income on health outcomes among men, such as CVD, using a quasi-experimental design.

Previous research on the health effects of public pension eligibility and receipt has observed mixed effects, although existing studies have used varying methodologies to evaluate a range of different health outcomes across heterogeneous populations and countries (2,10,12,15–17). Despite this inconsistency of research in other settings, there is strong plausibility for the OPG program in South Africa to benefit the health of older adults, including the reduction of hypertension risk. The OPG may reduce hypertension risk through various mechanisms operating in the short term, such as poverty alleviation and improved nutritional status, short- to medium term, such as increased utilization of healthcare services, and long term, such as improved mental health (18–22). Despite the economic benefits of the OPG, there is limited knowledge about its impact on the health of older adults in rural South Africa, particularly regarding hypertension (22). We thus aimed to investigate the effects of the South African OPG expansion on subsequent hypertension among older men who did and did not benefit from the expansion using data from a population-representative study of aging in rural northeast South Africa in 2014/2015.

To evaluate the impact of OPG on hypertension among older adults in rural South Africa, we employed 2 predictive tools for hypertension, each with its own strengths and limitations (23,24). The Framingham Heart Study hypertension risk prediction model (referred to hereafter as the “Framingham model”) is often used and well validated, whereas the Wand et al. hypertension risk prediction model was developed specifically for the South African context (23,24). The Framingham model was developed to identify individuals with an increased risk of hypertension in clinical settings and utilized data from White adult participants in the Framingham Heart Study (23). This model includes the following components: age, systolic blood pressure (SBP), diastolic blood pressure (DBP), body mass index (BMI), smoking status, and parental history of hypertension, and an interaction between age and DBP (23). This model has been validated in American and European populations, but not in African populations (23,25–27). In contrast, the Wand et al. model was adapted from the Framingham model for the South African population, using the nationally representative National Income Dynamics Study (NIDS) (24). This model includes all of the components in the Framingham model, as well as education, marital status, waist-to-hip ratio, alcohol intake, and weekly exercise (24). However, this model is relatively new, and its validity outside of the NIDS sample is not yet established. Therefore, we used both models and compared their results to gain comprehensive insights into the impact of the OPG expansion on hypertension risk among older men in rural South Africa.

## Method

### Study Population and Setting

“Health and Aging in Africa: A Longitudinal Study of an INDEPTH Community in South Africa” (HAALSI) is a population-representative longitudinal cohort study of aging (*n* = 5 059) (28). The design and measures of HAALSI are harmonized with those of the U.S. Health and Retirement Study, as one of its International Partner Studies. The HAALSI study population is representative of its source population of the rural Agincourt subdistrict in Mpumalanga province, in northeast South Africa. Eligible participants were men and women aged ≥40 as of July 1 2014, who had lived in Agincourt for at least 1 year prior to sampling. The HAALSI sample was identified and recruited through the Agincourt Health and Sociodemographic Surveillance System as its sampling frame (29). During Apartheid, Agincourt was designated as a region of forced residential segregation, called a “homeland,” for the Black South Africans belonging to the Shangaan ethnic group. During the Apartheid era, this region lacked basic health, education facilities, and living standards (11,12). Although living standards have improved post-Apartheid, Agincourt remains a low-income region with gaps in basic services such as tarred roads, piped water, and electricity. Baseline study interviews were conducted from November 2014 through November 2015, with follow-ups conducted every 3 years. Each in-person study interview included trained local fieldworker-administered questionnaires in the local Shangaan language using computer-assisted personal interviewing, as well as assessments of physical and cognitive function and biomarkers such as blood pressure (28).

The current analysis used cross-sectional baseline data. We excluded (i) women, as they were not eligible for the pension expansion policy (*n* = 2 714), (ii) men born before 1928, as they turned 65 prior to when the OPG became available to non-White South Africans in 1993 (*n* = 70), and (iii) men born after 1954, as they were not yet eligible for the OPG by the time of the study interview (*n* = 1 028). This resulted in a sample of 1 247 men aged ≥60 in the population-representative HAALSI in 2014/2015. We restricted the analysis to the cross-sectional baseline data to cleanly identify birth cohorts of men with specific durations of pension eligibility by the time of data collection. Finally, we excluded (iv) individuals with missing data on smoking status (*n* = 2), education status (*n* = 4), employment status (*n* = 14), waist-to-hip ratio (*n* = 83), and exercise (*n* = 5).

### OPG Expansion Eligibility

The main exposure variable was an ordinal measurement of the duration of OPG expansion eligibility (in years) at the time of data collection, ranging from 0 years through 5 years of additional pension expansion eligibility. We leveraged age-based exogenous variation in OPG expansion eligibility among men aged ≥60 at the time of their study interview. We combined information on birth year (ranging from 1928 to 1954) and year of the HAALSI interview (2014 or 2015) to group men into birth cohorts representing eligibility for 0 years of expanded OPG (ie, those who were aged ≥65 prior to 2008 when the expansion began, referred to as the control cohort), 1 additional year of OPG (ie, those who became eligible at age 64), 2 additional years of OPG (ie, those who became eligible at age 63), 3 additional years (ie, those who became eligible at age 62), 4 additional years of OPG (ie, those who became eligible at age 61), and 5 additional years of OPG (ie, those who became eligible at age 60). Table 1 contains full details of the OPG expansion eligibility classification.

**Table 1.** Description of OPG Expansion Cohorts by Additional Years of Pension Expansion Eligibility, HAALSI, 2014/2015 (*n* = 1 247)

Cohorts	Description	Birth Cohorts	*N* (%)
Control	Not eligible for OPG expansion (0 years)	1928–1943	577 (46.27)
1	Eligible for 1 additional year of OPG expansion	1944, 1953 (in 2014), 1954 (in 2015)	103 (8.26)
2	Eligible for 2 additional years of OPG expansion	1945, 1946, 1952 (in 2014), 1953 (in 2015)	151 (12.11)
3	Eligible for 3 additional years of OPG expansion	1947, 1951 (in 2014), 1951 (in 2015)	141 (11.31)
4	Eligible for 4 additional years of OPG expansion	1948–1950 (in 2014), 1951 (in 2015)	193 (15.48)
5	Eligible for 5 additional years of OPG expansion	1950 (in 2015)	82 (6.58)

*Notes*: HAALSI = Health and Aging in Africa: A Longitudinal Study of an INDEPTH Community in South Africa; OPG = Older Person’s Grant.

### Hypertension

Our outcome was a continuous measurement of the probability of hypertension (range: 0–1). There were no data on hypertension prior to the enrollment on the OPG, because these data came from a cohort study established after the OPG expansion concluded. Hypertension was identified from measurements of SBP and DBP taken by trained fieldworkers during the study interviews. To achieve precise blood pressure measurements, the fieldworkers first asked participants to sit for 5 minutes, after which they measured each of DBP and SBP 3 times 2 minutes apart (28). The final DBP and SBP measurements were the average of the second and third readings. Fieldworkers used the *OMRON© Automatic blood pressure monitor M6W.* We classified hypertension as meeting 1 or more of the following broad criteria: (i) SBP ≥ 140 mmHg and/or DBP ≥ 90 mmHg, (ii) self-reported having ever been diagnosed hypertension by a doctor, and (iii) being on treatment with cardiac medications, such as Enalapril, Amlodipine, Atenolol, Carvedilol, Furosemide, Hydrochlorothiazide, Methyldopa, Spironolactone, NIFEdipine, Isosorbide Dinitrate, and Simvastatin (28).

### Covariates

We used both a modified version of the Framingham model (revised to exclude parental history of hypertension, as this information was not available in our dataset), and the Wand et al. model to obtain the probability of hypertension. We also removed SBP and DBP from both prediction models to prevent overadjustment bias, because blood pressure may be a mediator on the causal pathway between pension expansion eligibility and hypertension. Predictors of hypertension from the modified Framingham model (23) were assessed in the in-person study interview: age (continuous, in years), smoking status (categorical, never or former smoker, current smoker), and BMI (continuous, kg/m^2^). Height and weight were measured in centimeters and kilograms, respectively, and were used to create measures of BMI. Additional predictors of hypertension from the Wand et al. model were also assessed in the in-person study interview: education (no formal education, 1–7 years, 8+ years), marital status (never married, currently married or living with a partner, separated or deserted, divorced, or widowed), alcohol consumption status (never consumed alcohol, ever consumed alcohol), waist-to-hip ratio (continuous, cm/cm), and weekly exercise (continuous, the sum of moderate, vigorous, walking, or bicycling hours per week) (24). Waist and hip circumferences were measured in centimeters with participants in the standing position, and were used to create measures of waist-to-hip ratio. There were 101 missing observations for BMI (8.1%) in our study sample. Because estimates are likely to be biased when more than 5% of observations are missing (30), we employed multiple imputations for analysis using both hypertension prediction models to compensate for the potential bias due to missing data.

### Statistical Analysis

First, descriptive characteristics of the sample were reported using the mean and standard deviation for continuous variables and the frequency and percentage for categorical variables. Consistent with Jock et al., we employed a multistep modeling approach to investigate the impact of each additional year of OPG eligibility on the probability of hypertension (31). All of the steps following were conducted using first the modified Framingham, and then the Wand et al. hypertension risk prediction models. We first developed a multivariable-adjusted logistic regression model to estimate the predicted probabilities of hypertension among men who were not eligible for the OPG expansion (ie, the control cohort) based on the hypertension predictors from each of the Framingham and the Wand et al. hypertension risk prediction models. The predicted probabilities estimated in these models were treated as the predicted probabilities of hypertension in the absence of OPG eligibility, conditional on known predictors of hypertension. We refer to these predicted probabilities as the “counterfactual” hypertension probabilities, as they represent the potential hypertension probability outcome for men in the expansion cohorts, had they not been exposed to expanded pension eligibility. We then developed a second multivariable-adjusted logistic regression model to estimate the predicted probabilities of hypertension among all men in the study sample. We refer to these predicted probabilities as the “observed” hypertension probabilities. Both “counterfactual” and “observed” hypertension probabilities are conditional on the modified Framingham Heart Study or the modified Wand et al. hypertension prediction model variables, and they differ in that the former are estimated in the control cohort, in the absence of OPG expansion, and the latter are estimated in the full study sample, with a range of OPG expansion exposures. We evaluated discrimination of the models using the area under the receiver-operating characteristic (ROC) curve. We also assessed multicollinearity in the prediction models using the variance inflation factor (VIF) statistic. Next, we computed the difference between the observed and counterfactual predicted probabilities of hypertension for men in the expansion cohorts, which indicated the difference in hypertension probabilities attributable to the OPG expansion. Finally, we estimated a linear regression model on the difference scores with an indicator for expansion cohort as a predictor, to determine the mean hypertension probability difference score for each cohort.

### Sensitivity Analyses

Because we used year of birth to identify OPG expansion eligibility and there are known age differences in hypertension risk (which are the same as birth cohort differences in this cross-sectional study design), we conducted a negative control analysis to evaluate the degree to which age differences may be influencing our results. We re-ran our analyses among women born between 1928 and 1954 (*n* = 1 346) taking consideration that women were not eligible for the OPG expansion but would presumably have been subjected to similar environmental exposures that affect hypertension risk as men (32). Additionally, existing evidence consistently suggests that women display a stronger association between age and hypertension risk than men (6). Therefore, if the negative control analysis among women shows a similar trend in difference scores as among men, it would imply that the observed effect among men could be attributable to age differences in hypertension risk rather than a true effect of the OPG expansion on hypertension (32). Similar to men, there were 117 (8.7%) missing observations for BMI among women; we employed multiple imputations for analysis using both hypertension prediction models to compensate for the potential bias due to missing data.

In addition, to assess whether there were any substantial differences in the results obtained, we performed a sensitivity analysis with the modified Framingham and Wand et al. models excluding the use of multiple imputations among men.

Furthermore, we conducted a sensitivity analysis using a prediction model limited to sociodemographic predictors of hypertension. The objective of this sensitivity analysis was to ensure that the sociodemographic model did not include any potential mediators on the hypothesized causal pathway between expanded pension eligibility and hypertension, thus avoiding any potential overadjustment. We developed the sociodemographic model using variables that would not plausibly be influenced by the OPG: age (continuous, in years), education (no formal education, 1–7 years, 8+ years), marital status (never married, currently married or living with a partner, separated or deserted, divorced, or widowed), household asset index quintile (continuous), employment status (unemployed, employed, homemaker), country of birth (born in South Africa, born outside of South Africa), and literacy (able to read and/or write, unable to read and/or write). We did not employ multiple imputations for analysis using this sociodemographic model because all the covariates had less than 5% of observations missing.

Finally, we executed a sensitivity analysis excluding men who were eligible for the pension for one year or less (ie, birth cohorts 1944 and 1953 with study interviews in 2014, and the 1954 birth cohort with interviews in 2015, *N* = 98).

Data cleaning procedures were conducted using StataSE 17 (College Station, TX). Data analyses and visualizations were conducted using R software v4.2.1 (R Foundation for Statistical Computing, Boston, MA).

## Results

The final analytical study sample was 1 139 men for analysis with the modified Framingham model, and 1 113 men for analysis with the Wand et al. model. Characteristics of the sample by OPG expansion cohort are shown in Table 2. The mean age was 70 years (range: 60–86 years). Of these men, 46% (*n* = 577) were not eligible for the OPG expansion and served as controls for this analysis, whereas 54% (*n* = 670) were eligible and belonged to 1 of the 5 expansion cohorts. The cohort with 4 years of additional OPG (*n* = 193, 29%) was the largest, whereas the cohort with 5 years of additional OPG eligibility was the smallest (*n* = 82, 12.2%). The prevalence of hypertension was high across all cohorts, ranging from 60% in the 1-year expansion cohort to 68% in the 3-year expansion cohort, with no discernible pattern across cohorts (Table 2).

**Table 2.** Characteristics of the Sample, Overall, and by OPG expansion Cohort, Men, HAALSI, 2014/2015 (*n* = 1 247)

Characteristic	OPG Expansion Cohort							
	Full Sample (*n* = 1 247)	Zero Years (*n* = 577)	One Year (*n* = 103)	Two Years (*n* = 151)	Three Years (*n* = 141)	Four Years (*n* = 193)	Five Years (*n* = 82)	*p* Value
Hypertension, *n* (%)								
No	410 (33.58)	180 (32.03)	41 (39.81)	47 (31.54)	43 (31.62)	70 (36.46)	29 (36.71)	.554^‡^
Yes	811 (66.42)	382 (67.97)	62 (60.19)	102 (66.46)	93 (68.38)	122 (63.54)	50 (63.29)	
Age in years, *M* (*SD*)	69.77 (7.23)	76.19 (5.13)	64.04 (4.86)	65.08 (3.74)	63.62 (2.49)	64.39 (1.25)	63.78 (0.44)	≤.001*
Smoking status, *n* (%)								
Former or never smoker	1,077 (86.37)	529 (91.68)	82 (79.61)	127 (84.11)	116 (82.27)	160 (82.90)	63 (76.83)	≤.001^‡^
Current smoker	170 (13.63)	48 (8.32)	21 (20.39)	24 (15.89)	25 (17.73)	33 (17.10)	19 (23.17)	
Body mass index, *M* (*SD*)	24.86 (5.46)	24.68 (5.45)	25.58 (5.15)	24.96 (5.64)	25.02 (5.58)	25.21 (5.40)	23.90 (5.43)	.302^†^
Education status, *n* (%)								
No education	663 (53.34)	356 (61.91)	42 (41.18)	56 (37.33)	64 (45.39)	99 (51.30)	46 (56.10)	≤.001^‡^
1–7 years	454 (36.52)	179 (31.13)	42 (41.18)	74 (49.33)	60 (42.55)	71 (36.79)	28 (34.15)	
8+ years	126 (10.14)	40 (6.96)	18 (17.65)	20 (13.33)	17 (12.06)	23 (11.92)	8 (9.76)	
Marital status, *n* (%)								
Never married	23 (1.84)	9 (1.56)	1 (0.97)	3 (1.99)	5 (3.55)	4 (2.07)	1 (1.22)	.101^‡^
Separated or deserted	71 (5.69)	23 (3.99)	5 (4.85)	11 (7.28)	10 (7.09)	13 (6.74)	9 (10.98)	
Divorced or widowed	234 (18.77)	130 (22.53)	13 (12.62)	25 (16.56)	24 (17.02)	28 (14.51)	14 (17.07)	
Currently married or living with partner	919 (73.70)	415 (71.92)	84 (81.55)	112 (74.17)	102 (72.34)	148 (76.68)	58 (70.73)	
Alcohol consumption status, *n* (%)								
Never consumed alcohol	388 (31.11)	180 (31.20)	35 (33.98)	53 (35.10)	33 (23.40)	64 (33.16)	23 (28.05)	≤.291^‡^
Ever consumed alcohol	859 (68.89)	397 (68.80)	68 (66.02)	98 (64.90)	108 (76.60)	129 (66.84)	59 (71.95)	
Waist-to-hip ratio, *M* (*SD*)	0.934 (0.075)	0.938 (0.081)	0.928 (0.077)	0.936 (0.074)	0.935 (0.066)	0.931 (0.066)	0.922 (0.065)	.002*
Exercise (hours per week), *M* (*SD*)	9.186 (17.287)	7.474 (15.907)	11.001 (18.670)	8.384 (14.312)	12.311 (22.402)	11.432 (18.269)	9.762 (16.276)	≤.001*
Employment status, *n* (%)								
Not working	1,042 (83.69)	510 (88.39)	76 (73.79)	116 (77.33)	110 (78.01)	162 (83.94)	68 (83.95)	≤.001^‡^
Employed	85 (6.83)	16 (2.77)	15 (14.56)	15 (10.00)	18 (12.77)	16 (8.29)	5 (6.17)	
Homemaker	118 (9.48)	51 (8.84)	12 (11.65)	19 (12.67)	13 (9.22)	15 (7.77)	8 (9.88)	
Household asset index, *M* (*SD*)	0.06 (2.37)	–0.14 (2.27)	0.69 (2.86)	0.37 (2.44)	0.47 (2.44)	–0.05 (2.22)	–0.37 (2.28)	≤.001*
Country of birth, *n* (%)								≤.001^‡^
Mozambique or other	396 (31.76)	211 (36.57)	20 (19.42)	41 (27.15)	29 (20.57)	57 (29.53)	38 (46.34)	
South Africa	851 (68.24)	366 (63.43)	83 (80.58)	110 (72.85)	112 (79.43)	136 (70.47)	44 (53.66)	
Literacy, *n* (%)								
Cannot read and write	513 (41.14)	279 (48.44)	31 (30.10)	45 (29.80)	47 (33.33)	75 (38.86)	36 (43.90)	.001^‡^
Able to read and/or write	733 (58.78)	297 (51.56)	72 (69.90)	106 (70.20)	94 (66.67)	118 (61.14)	46 (56.10)	

*Notes*: HAALSI = Health and Aging in Africa: A Longitudinal Study of an INDEPTH Community in South Africa; OPG = Older Person’s Grant; *SD* = standard deviation.

*Analysis of variance (ANOVA).

^†^Kruskal–Wallis rank-sum tests.

^‡^Pearson chi-square test.

Table 3 presents the results of multivariable-adjusted logistic regression models for hypertension according to the modified Framingham model and the Wand et al. model, in the full study sample and in the controls only. For the modified Framingham model, age and BMI were significantly associated with hypertension (Table 3). For the Wand et al. model, each of age, BMI, and marital status were significantly associated with hypertension (Table 3). We observed acceptable discrimination for both models, as indicated by the areas under the ROC curve of 0.647 (95% confidence interval [CI]: 0.614–0.680) and 0.666 (95% CI: 0.633–0.698) for the prediction of hypertension in the full sample using the modified Framingham model and the Wand et al. prediction model, respectively. Additionally, we observed areas under the ROC curve of 0.635 (95% CI: 0.602–0.667) and 0.655 (95% CI: 0.622–0.688) for the prediction of hypertension among the controls using the modified Framingham model and the Wand et al. model, respectively (Table 3). The multicollinearity test showed VIF values below 5 for both models, suggesting that there were no severe multicollinearity issues.

**Table 3.** Multivariable-Adjusted Logistic Regression Models Used to Estimate the Probabilities of Hypertension Among Older Men Using the modified Framingham Model (*n* = 1 139) and the Modified Wand et al. Model (*n* = 1 113), HAALSI, 2014/2015

Characteristic	Adjusted OR for Hypertension			
	Observed Model*^,^^†^	95% CI	Counterfactual Model^**^^,^^††^	95% CI
Framingham model, *n*	1 139		504	
Age (per year)	1.021	1.003, 1.039	1.050	1.012, 1.089
Smoking status				
Former or never smoked	1.00	(ref)	1.00	(ref)
Currently smoking	0.962	0.672, 1.377	0.901	0.464, 1.749
Body mass index (per unit increase)	1.102	1.072, 1.133	1.095	1.050, 1.143
Wand et al. model, *n*	1 113		492	
Age (per year)	1.026	1.007,1.045	1.045	1.005, 1.088
Smoking status				
Former or never smoked	1.00	(ref)	1.00	(ref)
Currently smoking	0.918	0.628, 1.341	0.890	0.442, 1.794
Body mass index (per unit increase)	1.083	1.050, 1.117	1.092	1.039, 1.147
Education status				
No formal education	1.00	(ref)	1.00	(ref)
1–7 years	1.273	0.964, 1.682	1.221	0.790, 1.886
8+ years	1.163	0.739, 1.832	0.779	0.366, 1.657
Marital status				
Never married	1.00	(ref)	1.00	(ref)
Currently married or living with a partner	3.535	1.282, 9.744	5.721	1.054, 31.054
Separated or deserted	3.054	0.995, 9.368	2.266	0.342, 15.003
Divorced or widowed	2.743	0.966, 7.789	3.866	0.690, 21.671
Alcohol consumption status				
Never consumed	1.00	(ref)	1.00	(ref)
Ever consumed	1.184	0.889, 1.576	1.158	0.752, 1.783
Waist-to-hip ratio (per unit increase)	19.810	3.021, 129.919	11.519	0.840, 158.047
Exercise (hours per week)	0.999	0.991, 1.005	0.994	0.983, 1.005

*Notes*: CI = confidence interval; HAALSI = Health and Aging in Africa: A Longitudinal Study of an INDEPTH Community in South Africa; OR = odds ratio; ROC = receiver-operating characteristic.

*ROC area for Framingham model = 0.647 (95% CI: 0.614–0.680).

^**^ROC area for Wand et al. model = 0.635 (95% CI: 0.602–0.667).

^†^ROC area for Framingham model = 0.666 (95% CI: 0.633–0.698).

^††^ROC area for Wand et al. model = 0.655 (95% CI: 0.622–0.688).

**Figure 1.**
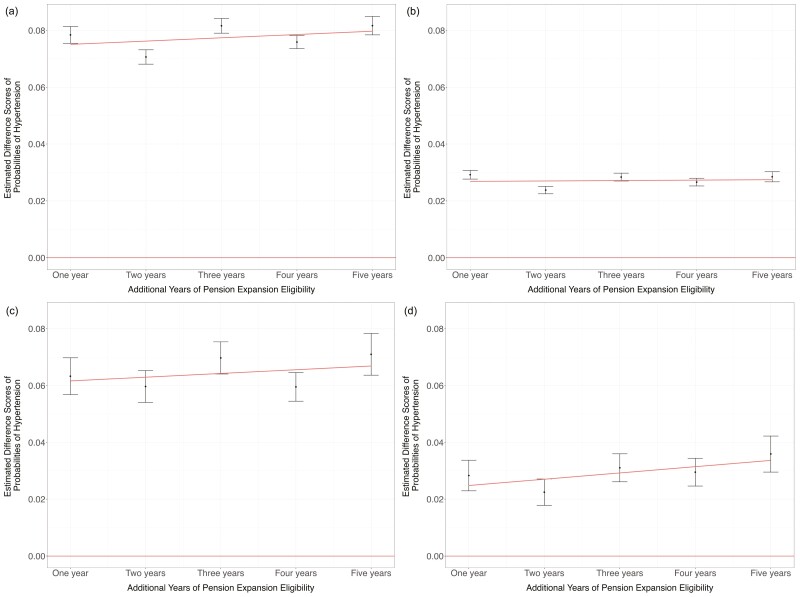
Comparison of estimated difference scores for the probability of hypertension in OPG expansion cohorts among men and women using Framingham and Wand et al. models, HAALSI, 2014/2015. (A) Estimated difference scores for the probability of hypertension for each OPG expansion cohort, men, HAALSI, 2014/2015, *N* = 1 139. Positive difference scores indicate a greater observed probability of hypertension than expected based on the modified Framingham model as applied in the control cohort who were not eligible for OPG expansion. (B) Negative control analysis of estimated difference scores for the probability of hypertension for each OPG expansion cohort using the modified Framingham model among women, HAALSI, 2014/2015, *N* = 1 225. (C) Estimated difference scores for the probability of hypertension for each OPG expansion cohort, men, HAALSI, 2014/2015, *N* = 1 113. Positive difference scores indicate a greater observed probability of hypertension than expected based on the Wand et al. model as applied in the control cohort who were not eligible for OPG expansion. (D) Negative control analysis of estimated difference scores for the probability of hypertension for each OPG expansion cohort using the modified Wand et al. model among women, HAALSI, 2014/2015, *N* = 1 197. HAALSI = Health and Aging in Africa: A Longitudinal Study of an INDEPTH Community in South Africa; OPG = = Older Person’s Grant.

Table 4 and Figure 1 (panels A and C) present the mean observed and counterfactual probabilities of hypertension based on the logistic regression models, and the difference scores for each OPG expansion cohort. We observed progressive but slight increases in the mean difference scores across expansion cohorts using the modified Framingham model (Table 4; Figure 1A). When using the modified Framingham model, the mean difference score for the 5-year expansion cohort was 0.081 (95% CI: 0.075, 0.088), indicating an 8.1 percentage point increase in the observed probability of having hypertension than expected in the absence of OPG expansion (Table 4). The linear line of best fit of the difference scores found a mean 0.16 percentage point increase in the probability of hypertension per additional year of pension eligibility, indicating a marginal to no dose–response impact of pension expansion across the expansion cohorts when using this model (Figure 1A). When using the Wand et al. model, we observed similar but slightly smaller difference scores for the probabilities of hypertension as with the Framingham model (Table 4). The mean difference score for the five-year expansion cohort was 0.069 (95% CI: 0.055, 0.084) when using the Wand et al. model, indicating a 6.9 percentage point increase in the observed probability of having hypertension than expected in the absence of OPG expansion (Table 4). The linear line of best fit of the difference scores found a mean 0.03 percentage point increase in the probability of hypertension per additional year of pension eligibility, indicating no dose–response impact of pension expansion across the cohorts with increasing duration of exposure to pension expansion when using the modified Wand et al. model (Figure 1C).

**Table 4.** Mean Observed and Counterfactual Predicted Probabilities of Hypertension and Difference Scores Among Men in the Control and 5 Expansion Cohorts Using the Modified Framingham Model (*n* = 1 139) and the Wand et al. model (*n* = 1 113), HAALSI, 2014/2015

Pension Expansion Cohorts	Mean Predicted Probabilities of Hypertension (*SD*)		Difference (*SD*) (observed—counterfactual)	Difference Score (95% CI)	*p* Value
	Observed	Counterfactual			
Framingham model, *n*	1 139	504			
0 years, control	0.692 (0.004)	0.678 (0.005)	0.014 (0.001)	—	—
1 additional year	0.645 (0.011)	0.553 (0.013)	0.092 (0.004)	0.079 (0.073, 0.085)	≤.001
2 additional years	0.646 (0.009)	0.562 (0.011)	0.084 (0.002)	0.070 (0.065, 0.076)	≤.001
3 additional years	0.635 (0.009)	0.539 (0.010)	0.095 (0.002)	0.081 (0.076, 0.087)	≤.001
4 additional years	0.643 (0.008)	0.554 (0.009)	0.089 (0.001)	0.075 (0.071, 0.080)	≤.001
5 additional years	0.623 (0.012)	0.527 (0.013)	0.095 (0.001)	0.081 (0.075, 0.088)	≤.001
Wand et al. model, *n*	1 113	492			
0 years, control	0.695 (0.005)	0.685 (0.006)	0.010 (0.002)	—	—
1 additional year	0.638 (0.013)	0.564 (0.016)	0.074 (0.007)	0.063 (0.050, 0.076)	≤.001
2 additional years	0.650 (0.011)	0.580 (0.014)	0.070 (0.006)	0.060 (0.049, 0.071)	≤.001
3 additional years	0.632 (0.011)	0.551 (0.015)	0.080 (0.005)	0.070 (0.059, 0.081)	≤.001
4 additional years	0.636 (0.010)	0.566 (0.012)	0.070 (0.004)	0.060 (0.050, 0.070)	≤.001
5 additional years	0.608 (0.016)	0.529 (0.020)	0.080 (0.008)	0.069 (0.055, 0.084)	≤.001

*Notes*: CI = confidence interval; HAALSI = Health and Aging in Africa: A Longitudinal Study of an INDEPTH Community in South Africa; *SD* = standard deviation.

Table 5 shows the results of the negative control analysis among women. The analytical cohort for the negative control analysis using the modified Framingham model analysis consisted of 1 225 older women after excluding 4 (0.30%) with missing data on at least 1 of the 3 covariates for this model. The analytical cohort for the negative control analysis using the Wand et al. model consisted of 1 197 older women after excluding 14 (1.04%) with missing data on at least one of the covariates for this model. Missing values of BMI were imputed for both models. We observed nonzero difference scores for each expansion cohort in this analysis which were statistically significant, although there was no evidence for a linear dose–response trend in difference scores across expansion cohorts (Figure 1B and D). When using the modified Framingham model, the mean difference score for the 5-year expansion cohort was 0.029 (95% CI: 0.025, 0.032), indicating that a 2.9 percentage point difference between the observed and predicted hypertension probability is likely due to age differences (Table 5). Similarly, when using the Wand et al. model, the mean difference score for the 5-year expansion cohort was 0.039 (95% CI: 0.026, 0.052), indicating that a 3.9 percentage point difference between the observed and predicted hypertension probability is likely due to age differences (Table 5). When these estimates are subtracted from those from the main analysis among men, the results are reduced to a 5.2 percentage point difference when using the Framingham model and a 3.0 percentage point difference when using the Wand et al. model.

**Table 5.** Negative Control Analysis of the Mean Observed and Counterfactual Predicted Probabilities of Hypertension and Differences Scores Among Women Using the Modified Framingham Model (*n* = 1 225) and the Wand et al. Model (*n* = 1 197), HAALSI 2014/2015

Negative Control Expansion Cohorts	Mean Predicted Probabilities of Hypertension (*SD*)		Difference (*SD*) (observed—counterfactual)	Difference Score (95% CI)	*p* Value
	Observed	Counterfactual			
Framingham model, *n*	1 225	525			
0 years, control	0.768 (0.003)	0.765 (0.004)	0.003 (0.001)	—	—
1 additional year	0.711 (0.009)	0.679 (0.010)	0.032 (0.002)	0.028 (0.025, 0.031)	≤.001
2 additional years	0.728 (0.007)	0.702 (0.008)	0.026 (0.001)	0.023 (0.020, 0.025)	≤.001
3 additional years	0.721 (0.008)	0.691 (0.009)	0.031 (0.001)	0.027 (0.025, 0.030)	≤.001
4 additional years	0.725 (0.008)	0.697 (0.009)	0.029 (0.001)	0.025 (0.023, 0.028)	≤.001
5 additional years	0.712 (0.011)	0.680 (0.013)	0.032 (0.002)	0.029 (0.025, 0.032)	≤.001
Wand et al. model, *n*	1 197	497			
0 years, control	0.769 (0.004)	0.766 (0.005)	0.004 (0.002)	—	—
1 additional year	0.706 (0.011)	0.675 (0.014)	0.031 (0.005)	0.027 (0.016, 0.038)	≤.001
2 additional years	0.727 (0.009)	0.701 (0.012)	0.026 (0.004)	0.022 (0.013, 0.031)	≤.001
3 additional years	0.717 (0.010)	0.683 (0.014)	0.035 (0.005)	0.031 (0.021, 0.040)	≤.001
4 additional years	0.720 (0.010)	0.686 (0.013)	0.034 (0.005)	0.030 (0.020, 0.039)	≤.001
5 additional years	0.718 (0.013)	0.674 (0.016)	0.043 (0.007)	0.039 (0.026, 0.052)	≤.001

*Notes*: CI = confidence interval; HAALSI = Health and Aging in Africa: A Longitudinal Study of an INDEPTH Community in South Africa; *SD* = standard deviation.

In the sensitivity analysis without multiple imputations for missing values of BMI, we observed similar results and consistent trends in difference scores across expansion cohorts when using both the Framingham and Wand et al. models (see Supplementary Tables 1 and 2). In the sensitivity analysis using the sociodemographic prediction model, the results were consistent with the main analysis, although the areas under the ROC curve for predicting hypertension were weaker than when using the Framingham or Wand et al. models and the difference scores were slightly weaker in magnitude than when using either of these models (Supplementary Tables 3 and 4). Finally, we observed similar results to our main results when we executed a sensitivity analysis excluding men who were eligible for the pension for 1 year or less (see Supplementary Materials 5 and 6).

## Discussion

In this large, population-based study in a rural region of South Africa, we examined the relationship between exposure to expanded OPG eligibility and the probability of hypertension among older men. We demonstrated that exposure to expanded pension eligibility is associated with higher probabilities of hypertension in this population. Exposure to 5 additional years of pension eligibility, the longest exposure duration under study, was associated with a 6.9–8.1 percentage point increase in the probability of hypertension among older men. Because there were approximately 4 000 men aged 60 years or older living in the study region in 2019, these effect sizes correspond to approximately 276–324 additional men having hypertension after an additional 5 years of OPG eligibility in this setting (33). If these results are conservatively revised to account for the birth cohort effects identified in our negative control analysis, the 3.0–5.2 percentage point increase in hypertension probability would correspond to an approximate 120–208 additional men with hypertension than expected in the absence of OPG expansion. Although this result may indicate a large impact of the OPG expansion on the population hypertension burden, it requires confirmation in other population groups and geographic regions of South Africa, and over a longer follow-up time frame for long-term policy evaluation. In addition, the important social, economic, and other health benefits of expanded OPG access among low-income older adults should be considered holistically alongside these results when evaluating its impact on the population (16,34–36).

We offer 2 potential explanations for the marginal increase in hypertension probability that we observed among older men with increasing additional years of pension eligibility. The first is that greater income may result in worse cardiovascular-related behaviors and outcomes, consistent with other evidence from South Africa (37,38). In recent years, many low- and middle-income countries have experienced rapid economic growth, resulting in an increase in dietary consumption of sugar, lipids, and processed carbohydrates (39). Additional pension income may promote expenditure on high-calorie foods leading to an increase in the probability of hypertension (40–42). If this is the case, then health education along with pension income transfer may help to promote cardiovascular health among older adults. Indeed, a number of studies in middle-income countries have demonstrated that cash transfer programs, when conditional on health behaviors and uptake of educational services by mothers, have resulted in improved health outcomes for their infants, as compared to cash transfer programs without any participation requirements (43–46). In this study context, pairing pension provision with hypertension health education could be particularly valuable. Our data identified that 41.69% of men in the analytical sample reported that a health professional had diagnosed them with hypertension. Although hypertension awareness, treatment, and control have been improving in Agincourt over time (47), there remain gaps at each point of the treatment cascade, especially for men. Moreover, another study conducted within Agincourt has demonstrated the feasibility of implementing a health education intervention at the point of OPG delivery to reduce sodium intake and improve blood pressure control (19). Although this was a small feasibility study, it indicates that health education interventions provided alongside the South African pension program could be feasible in improving cardiovascular health among older adults.

The second potential explanation is that the pension alone may not be a sufficient source of income to meaningful changes to lifestyle and health that would reduce cardiovascular risk. In previous studies in Brazil and Mexico, cash transfers of similar amount to that of the OPG were found to have a marginally protective or negligible effect on cardiovascular disease onset and mortality (16,48). Indeed, a study in the same rural region of South Africa, also using the HAALSI data, identified no effect of the OPG expansion on physical disabilities among older adults (49). Any effect of the OPG expansion on hypertension is likely to depend on what pension beneficiaries do with their additional pension income, and whether this additional income affects blood pressure through indirect, nonexpenditure pathways, such as altering health-related behaviors or levels of stress. Previous research has found older adults in rural South Africa spend a significant portion of their pension funds to subsidize their food purchases (9,41,50). However, pension spending may be not uniformly used for the purchase of health-promoting goods. Although a growing number of studies indicate that concerns regarding the use of cash transfers to buy alcohol and tobacco are unwarranted (51), a recent study found that alcohol and tobacco accounted for >40% of food and drink consumption among pension-receiving households in the present study region of South Africa (12). Men in sub-Saharan Africa have a larger propensity to use alcohol and tobacco than women, which likely contributes to the growing cardiovascular disease burden among men in this global region (52,53). Future studies should investigate pension spending practices in rural South Africa pertaining to both health-harming and health-promoting goods and behaviors, to better understand the net effect of pensions on health outcomes.

This study has limitations. First, both the Framingham and Wand et al. models contain potential mediators of the effect of pension eligibility on hypertension, such as BMI (34,54)^.^ When we conducted a sensitivity analysis using a prediction model that included only sociodemographic factors without potential mediators, we observed results of a weaker magnitude. This attenuation could be because the sociodemographic model did not perform as well as the Framingham and Wand et al. models in predicting hypertension, but it may also be that the mediating pathways from pension exposure to hypertension could act in conflicting directions that lead to an unpredictable direction of overadjustment bias. Previous evidence from low-income settings shows conflicting findings regarding the effect of cash transfers on health behaviors, such as substance use and exercise (34,35,55,56), suggesting that the pathways linking pension income to hypertension may operate in opposite directions. We also did not have data on parental history of hypertension to use in our hypertension probability model, which may have reduced the discriminatory capability of our model (28). In addition, we only had a binary alcohol consumption measure, which may be subject to reporting error or bias that could have affected the predictive capability of the Wand et al. model used in this analysis (57). Future studies should address how to minimize error and biases in the measurement of alcohol consumption in this study context.

The iterative OPG expansions in 2008, 2009, and 2010 were implemented in April of each year (14), although we rounded up the durations of expanded pension eligibility to full years. Therefore, older men who became eligible for the OPG during these years but with birthdays before April received 1–4 months fewer than a full year of pension eligibility (*N* = 471). Because the birth month is random, we expect that this exposure misclassification is nondifferential with respect to the exposure and outcome and may have biased our results to the null. Furthermore, the duration of expanded pension eligibility is rounded up to a full year for all individuals, regardless of when their birthdates were with respect to their HAALSI study interview. However, 71% of the sample had their birthday within 4 months of their HAALSI interview. We also expect that this source of measurement error would be nondifferential with respect to the exposure and outcome, and, if anything, it may have biased our results to the null.

Moreover, we may not have been able to observe the full, long-term effect of the OPG expansion on hypertension in this study. Although there is evidence that lifestyle interventions can alter blood pressure in as little as 6 months, it is unclear whether the same delay would be seen when applied to pension income (58,59). In addition, at the time of the study interview, several of those exposed to the OPG expansion were pension recipients for a brief period—less than a year. However, we found similar results when we conducted a sensitivity analysis excluding men exposed to the OPG expansion for 1 year or less. We did not conduct a longitudinal analysis due to the intricacies of men continually aging into pension eligibility over the follow-up period. Future research should use long-term longitudinal data to investigate the health effects of accumulated pension receipt over extended periods, using hypertension as well as other cardiovascular health outcomes to gain a comprehensive picture of the impact of the OPG on the health of older adults.

Additionally, we used pension eligibility rather than pension receipt as our exposure variable. The use of pension eligibility rather than pension receipt as the exposure variable represents an intention-to-treat (ITT) estimate, which has a greater policy relevance as the minimum effect of the OPG on hypertension among older men in rural South Africa. Although the majority of those eligible for pensions in our study region received their pension payments, it is important to note that the pension uptake was relatively low among men immediately following the implementation of the pension expansion policy (22,60). Because the ITT estimate gives the effect of being assigned to receive the treatment, as pension uptake declines, the difference between the effect of being assigned to pension expansion and the effect of actually receiving expanded pension increases, leading to an attenuation of the ITT estimate compared to the true causal effect (61,62). Nevertheless, the ITT analysis is regarded as the most robust method for assessing the impact of policies on health outcomes (61,62).

Finally, it is crucial to recognize the influence of other social protection grants in South Africa. Hypertension is a qualifying condition for South Africa’s public disability grant, which individuals can receive in the years prior to becoming pension eligible. For this population group, aging into pension eligibility would have signified a change in eligibility from the disability grant to the OPG. Aging into pension eligibility thus may not materially change income for those individuals who receive disability grants, indicating that there may be heterogeneous effects of OPG eligibility on hypertension probability across the general population. Additional research should explore the complex interactions between South Africa’s rich social protection grant schemes on hypertension, as well as other aging-relevant outcomes.

This study has numerous strengths. We employed a large and population-representative sample of older men in an underrepresented, low-income region of rural South Africa. We used objectively measured hypertension as the outcome, as opposed to self-reported data, which reduces measurement error and recall bias. We used birth year as an indicator of exogenous variation in additional eligibility for OPG income, leveraging a natural experimental approach. Although birth year as the source of exogenous variation in the exposure introduces the possibility of age differences or birth cohort effects (which are the same in this study design), using data from women in a negative control analysis helped to confirm the robustness of our findings.

In conclusion, we found that the noncontributory OPG alone may not be sufficient to improve cardiovascular health among rural South African men. Indeed, we observed marginal increases in the likelihood of hypertension among men who benefitted from the OPG. Future studies should investigate how pension funds are managed and spent among older adults in rural South Africa and other regions, and how health education interventions may be effectively tied to pension receipt to maximize their potential for positive population health impact. As populations continue to age, policy-makers must consider how social protection grants such as pensions may be best implemented to both financially protect older adults and promote their healthy aging. These findings add to the policy conversation by highlighting the potential link between pensions and hypertension among older men in rural South Africa and areas for future research on this topic.

## Supplementary Material

igae010_suppl_Supplementary_Table_S1-S6
