# Supplementary material for: The Impact of the Older Person’s Grant Expansion on Hypertension Among Older Men in Rural South Africa: Findings From the HAALSI Cohort
Source: Innov Aging. 2024 Feb 8;8(4):igae010. doi: 10.1093/geroni/igae010 (PMC11020309; doi:10.1093/geroni/igae010)
Supplement: igae010_suppl_Supplementary_Table_S1-S6 [file igae010_suppl_supplementary_table_s1-s6.docx]

*Innovation in Aging* Supplementary Material: Chang, Haeyoon; Jock, Janet; Rosenberg, Molly; Li, Chihua; Cho, Tsai-Chin; Lisabeth, Lynda; Gaziano, Thomas; Kobayashi, Lindsay. The Impact of the Older Person’s Grant Expansion on Hypertension Among Older Men in Rural South Africa: Findings from the HAALSI cohort.

**Supplemental Table 1. Multivariable-adjusted logistic regression models used to estimate the probabilities of hypertension among older men using the modified Framingham (n=1,139) and the Wand et al. (n=1,113) hypertension risk prediction model without multiple imputation, HAALSI 2014/15.**

| **Characteristic** | **Adjusted OR for hypertension** | | | |
| --- | --- | --- | --- | --- |
| Framingham model | **Observed model^*^**  **(n=1,139)** | **95% CI** | **Counterfactual model^**^ (n=504)** | **95% CI** |
| Age (per year) | 1.029 | 1.010, 1.048 | 1.057 | 1.016, 1.099 |
| Smoking status |  |  |  |  |
| Former or never smoked | 1.00 | (ref) | 1.00 | (ref) |
| Currently smoking | 0.977 | 0.673, 1.418 | 0.912 | 0.446, 1.865 |
| BMI (per unit increase) | 1.118 | 1.085, 1.151 | 1.132 | 1.078, 1.188 |
|  |  |  |  |  |
| Wand et al. model | **Observed model^†^ (n=1,113)** | **95% CI** | **Counterfactual model^††^ (n=492)** | **95% CI** |
| Age (per year) | 1.027 | 1.008,1.047 | 1.048 | 1.005, 1.093 |
| Smoking status |  |  |  |  |
| Former or never smoked | 1.00 | (ref) | 1.00 | (ref) |
| Currently smoking | 0.914 | 0.620, 1.346 | 0.877 | 0.423, 1.820 |
| BMI (per unit increase) | 1.092 | 1.057, 1.129 | 1.113 | 1.055, 1.173 |
| Education status |  |  |  |  |
| No formal education | 1.00 | (ref) | 1.00 | (ref) |
| 1-7 years | 1.233 | 0.929, 1.636 | 1.136 | 0.726, 1.776 |
| 8 + years | 1.127 | 0.713, 1.782 | 0.685 | 0.318, 1.478 |
| Marital status |  |  |  |  |
| Never married | 1.00 | (ref) | 1.00 | (ref) |
| Currently married or living with a partner | 3.224 | 1.148, 9.053 | 4.142 | 0.714, 24.018 |
| Separated or deserted | 2.890 | 0.923, 9.046 | 1.968 | 0.279, 13.896 |
| Divorced or widowed | 2.598 | 0.896, 7.532 | 2.967 | 0.494, 17.822 |
| Alcohol consumption status |  |  |  |  |
| Never consumed | 1.00 | (ref) | 1.00 | (ref) |
| Ever consumed | 1.163 | 0.870, 1.557 | 1.175 | 0.753, 1.834 |
| Waist-to-hip ratio (per unit increase) | 17.095 | 2.538, 115.148 | 10.303 | 0.716, 148.188 |
| Exercise (hours per week) | 0.999 | 0.992, 1.006 | 0.995 | 0.983, 1.007 |

* ROC area = 0.656 (95% CI: 0.623-0.689), **ROC area = 0.650 (95% CI: 0.616-0.683), **^†^** ROC area = 0.667 (95% CI: 0.633-0.700), **^††^**ROC area = 0.656 (95% CI: 0.623-0.690).

**Supplemental Table 2. Mean observed and counterfactual predicted probabilities of hypertension and difference scores among men in the control and five expansion cohorts using the modified Framingham (n=1,139) and the Wand et al. (n=1,113) hypertension risk model without multiple imputation, HAALSI, 2014/15.**

| **Pension Expansion Cohorts** | **Mean predicted probabilities of hypertension (SD)** | | **Difference (SD)**  **(Observed – Counterfactual)** | **Difference Score (95% CI)** | **p-value** |
| --- | --- | --- | --- | --- | --- |
| Framingham model | **Observed (n=1,139)** | **Counterfactual (n=504)** |  |  |  |
| Zero years, control | 0.701 (0.104) | 0.689 (0.124) | 0.011 (0.031) | - | - |
| One additional year | 0.638 (0.121) | 0.555 (0.152) | 0.083 (0.039) | 0.072 (0.065, 0.079) | ≤ 0.001 |
| Two additional years | 0.639 (0.126) | 0.563 (0.157) | 0.076 (0.036) | 0.065 (0.059, 0.070) | ≤ 0.001 |
| Three additional years | 0.625 (0.119) | 0.538 (0.144) | 0.087 (0.029) | 0.076 (0.070, 0.082) | ≤ 0.001 |
| Four additional years | 0.636 (0.119) | 0.555 (0.143) | 0.081 (0.026) | 0.069 (0.064, 0.075) | ≤ 0.001 |
| Five additional years | 0.612 (0.123) | 0.523 (0.145) | 0.089 (0.024) | 0.077 (0.070, 0.085) | ≤ 0.001 |
|  |  |  |  |  |  |
| Wand et al. model | **Observed (n=1,113)** | **Counterfactual (n=492)** |  |  |  |
| Zero years, control | 0.698 (0.118) | 0.689 (0.140) | 0.010 (0.051) | - | - |
| One additional year | 0.635 (0.126) | 0.560 (0.165) | 0.075 (0.066) | 0.066 (0.053, 0.078) | ≤ 0.001 |
| Two additional years | 0.647 (0.132) | 0.578 (0.172) | 0.070 (0.068) | 0.060 (0.049, 0.071) | ≤ 0.001 |
| Three additional years | 0.629 (0.133) | 0.550 (0.170) | 0.079 (0.062) | 0.069 (0.058, 0.081) | ≤ 0.001 |
| Four additional years | 0.634 (0.133) | 0.564 (0.165) | 0.070 (0.060) | 0.060 (0.050, 0.070) | ≤ 0.001 |
| Five additional years | 0.605 (0.136) | 0.527 (0.173) | 0.078 (0.065) | 0.068 (0.054, 0.083) | ≤ 0.001 |

**Supplemental Table 3. Multivariable-adjusted logistic regression models used to estimate the probabilities of hypertension among older men using the sociodemographic hypertension risk prediction model, HAALSI 2014/15, n=1,214**

| **Characteristic** | **Adjusted OR for hypertension** | | | |
| --- | --- | --- | --- | --- |
| Sociodemographic Model | **Observed model**^‡^  **(n=1,214)** | **95% CI** | **Counterfactual model**^‡‡^  **(n=558)** | **95% CI** |
| Age | 1.027 | 1.009, 1.046 | 1.046 | 0.008, 1.085 |
| Education status |  |  |  |  |
| No formal education | 1.00 | (ref) | 1.00 | (ref) |
| 1-7 years | 1.045 | 0.759, 1.439 | 1.177 | 0.720, 1.923 |
| 8 + years | 0.947 | 0.575, 1.559 | 0.672 | 0.301, 1.503 |
| Marital status |  |  |  |  |
| Never married | 1.00 | (ref) | 1.00 | (ref) |
| Currently married or living with a partner | 3.179 | 1.246, 8.110 | 5.240 | 0.989, 27.771 |
| Separated or deserted | 2.126 | 0.752, 6.012 | 1.930 | 0.299, 12.434 |
| Divorced or widowed | 2.473 | 0.944, 6.477 | 3.495 | 0.642, 19.015 |
| Household asset index (per quintile increase) | 1.059 | 0.996, 1.126 | 1.061 | 0.954, 1.153 |
| Employment status |  |  |  |  |
| Not working | 1.00 | (ref) | 1.00 | (ref) |
| Employed | 1.464 | 0.870, 2.464 | 2.173 | 0.591, 7.991 |
| Homemaker | 1.229 | 0.802, 1.881 | 1.167 | 0.609, 2.238 |
| Country of birth |  |  |  |  |
| Mozambique or other | 1.00 | (ref) | 1.00 | (ref) |
| South Africa | 1.085 | 0.814, 1.446 | 1.162 | 0.773, 1.747 |
| Literacy |  |  |  |  |
| Unable to read and write | 1.00 | (ref) | 1.00 | (ref) |
| Able to read and/or write | 1.485 | 1.102, 2.002 | 1.328 | 0.855, 2.065 |
|  |  |  |  |  |

^‡^ROC area = 0.611 (95% CI: 0.577-0.644), ^‡‡^ROC area = 0.597 (95% CI: 0.563-0.630).

**Supplemental Table 4. Mean observed and counterfactual predicted probabilities of hypertension and difference scores among men in the control and five expansion cohorts using the sociodemographic hypertension risk model without multiple imputation, HAALSI, 2014/15, n=1,214**

| **Pension Expansion Cohorts** | **Mean predicted probabilities of hypertension (SD)** | | **Difference (SD)**  **(Observed – Counterfactual)** | **Difference Score (95% CI)** | **p-value** |
| --- | --- | --- | --- | --- | --- |
| Sociodemographic model | **Observed (n=1,214)** | **Counterfactual**  **(n=558)** |  |  |  |
| Zero years, control | 0.688 (0.082) | 0.677 (0.107) | 0.010 (0.046) | - | - |
| One additional year | 0.662 (0.093) | 0.609 (0.121) | 0.054 (0.055) | 0.043 (0.032, 0.055) | ≤ 0.001 |
| Two additional years | 0.655 (0.094) | 0.599 (0.129) | 0.056 (0.059) | 0.046 (0.036, 0.055) | ≤ 0.001 |
| Three additional years | 0.642 (0.098) | 0.582 (0.135) | 0.060 (0.062) | 0.050 (0.040, 0.060) | ≤ 0.001 |
| Four additional years | 0.634 (0.092) | 0.576 (0.124) | 0.058 (0.058) | 0.048 (0.039, 0.057) | ≤ 0.001 |
| Five additional years | 0.614 (0.086) | 0.545 (0.119) | 0.069 (0.061) | 0.059 (0.047, 0.071) | ≤ 0.001 |
|  |  |  |  |  |  |

**Supplemental Table 5. Multivariable-adjusted logistic regression models used to estimate the probabilities of hypertension among older men using the modified Framingham model (n=1,041) and Wand et al. model (n=1,017) excluding cohorts that received pension less than one year with multiple imputation, HAALSI 2014/15**

| **Characteristic** | **Adjusted OR for hypertension** | | | |
| --- | --- | --- | --- | --- |
| Framingham model | **Observed model^*^**  **(n=1,041)** | **95% CI** | **Counterfactual model^**^**  **(n=504)** | **95% CI** |
| Age (per year) | 1.029 | 1.010, 1.048 | 1.044 | 1.016, 1.072 |
| Smoking status |  |  |  |  |
| Former or never smoked | 1.00 | (ref) | 1.00 | (ref) |
| Currently smoking | 0.977 | 0.673, 1.418 | 1.121 | 0.621, 2.025 |
| BMI (per unit increase) | 1.118 | 1.085, 1.151 | 1.138 | 1.090, 1.188 |
|  |  |  |  |  |
| Wand et al. model | **Observed model^†^**  **(n=1,017)** | **95% CI** | **Counterfactual**  **model^††^ (n=492)** | **95% CI** |
| Age (per year) | 1.025 | 1.006,1.045 | 1.043 | 1.014, 1.072 |
| Smoking status |  |  |  |  |
| Former or never smoked | 1.00 | (ref) | 1.00 | (ref) |
| Currently smoking | 0.918 | 0.628, 1.341 | 1.122 | 0.620, 2.033 |
| BMI (per unit increase) | 1.083 | 1.050, 1.117 | 1.104 | 1.057, 1.155 |
| Education status |  |  |  |  |
| No formal education | 1.00 | (ref) | 1.00 | (ref) |
| 1-7 years | 1.273 | 0.964, 1.682 | 1.306 | 0.878, 1.941 |
| 8 + years | 1.163 | 0.739, 1.832 | 1.166 | 0.593, 2.294 |
| Marital status |  |  |  |  |
| Never married | 1.00 | (ref) | 1.00 | (ref) |
| Currently married or living with a partner | 3.535 | 1.282, 9.744 | 5.500 | 1.063, 28.466 |
| Separated or deserted | 3.054 | 0.995, 9.368 | 2.114 | 0.344, 12.986 |
| Divorced or widowed | 2.743 | 0.966, 7.789 | 3.496 | 0.656, 18.640 |
| Alcohol consumption status |  |  |  |  |
| Never consumed | 1.00 | (ref) | 1.00 | (ref) |
| Ever consumed | 1.184 | 0.889, 1.576 | 1.262 | 0.847, 1.879 |
| Waist-to-hip ratio (per unit increase) | 19.810 | 3.021, 129.919 | 24.649 | 2.088, 291.034 |
| Exercise (hours per week) | 0.998 | 0.991, 1.005 | 0.996 | 0.986, 1.006 |

* ROC area = 0.656 (95% CI: 0.623-0.689), **ROC area = 0.654 (95% CI: 0.620-0.687), **^†^** ROC area = 0.666 (95% CI: 0.633-0.698), **^††^**ROC area = 0.659 (95% CI: 0.626-0.692).

**Supplemental Table 6. Mean observed and counterfactual predicted probabilities of hypertension and difference scores among men in the control and five expansion cohorts using the modified Framingham model (n=1,041) and Wand et al. model (n=1,017) excluding cohorts that received pension for less than one year, HAALSI, 2014/15**

| **Pension Expansion Cohorts** | **Mean predicted probabilities of hypertension (SD)** | | **Difference (SD)**  **(Observed – Counterfactual)** | **Difference Score (95% CI)** | **p-value** |
| --- | --- | --- | --- | --- | --- |
| Framingham model | **Observed model**  **(n=1,041)** | **Counterfactual model**  **(n=504)** |  |  |  |
| Zero years, control | 0.691 (0.004) | 0.674 (0.005) | 0.017 (0.001) | - | - |
| One additional year | - | - | - | - | - |
| Two additional years | 0.639 (0.011) | 0.596 (0.013) | 0.043 (0.002) | 0.026 (0.022, 0.031) | ≤ 0.001 |
| Three additional years | 0.625 (0.010) | 0.577 (0.012) | 0.048 (0.002) | 0.031 (0.027, 0.036) | ≤ 0.001 |
| Four additional years | 0.636 (0.009) | 0.591 (0.010) | 0.045 (0.002) | 0.028 (0.024, 0.032) | ≤ 0.001 |
| Five additional years | 0.612 (0.014) | 0.563 (0.017) | 0.049 (0.003) | 0.032 (0.026, 0.038) | ≤ 0.001 |
|  |  |  |  |  |  |
|  | **Observed model**  **(n=1,017)** | **Counterfactual model**  **(n=492)** | **Difference (SD)**  **(Observed –Counterfactual)** | **Difference Score (95% CI)** | **p-value** |
| Wand et al. model |  |  |  |  |  |
| Zero years, control | 0.686 (0.005) | 0.670 (0.006) | 0.016 (0.002) | - | - |
| One additional year | - | - | - | - | - |
| Two additional years | 0.650 (0.011) | 0.605 (0.015) | 0.045 (0.005) | 0.029 (0.019, 0.039) | ≤ 0.001 |
| Three additional years | 0.632 (0.012) | 0.581 (0.015) | 0.051 (0.005) | 0.035 (0.025, 0.045) | ≤ 0.001 |
| Four additional years | 0.636 (0.010) | 0.590 (0.013) | 0.046 (0.004) | 0.030 (0.021, 0.039) | ≤ 0.001 |
| Five additional years | 0.608 (0.016) | 0.549 (0.021) | 0.059 (0.007) | 0.043 (0.029, 0.056) | ≤ 0.001 |
